# Using scanning electron microscopy (SEM) to study morphology and morphometry of the isolated haptoral sclerites of three distinct diplozoid species

**DOI:** 10.1371/journal.pone.0211794

**Published:** 2019-02-25

**Authors:** Quinton Marco Dos Santos, Ewa Dzika, Annemariè Avenant-Oldewage

**Affiliations:** 1 Department of Zoology, University of Johannesburg, Auckland Park, Johannesburg, South Africa; 2 Department of Medical Biology, Faculty of Medical Science, Warmia and Mazury University, Olsztyn, Poland; Fred Hutchinson Cancer Research Center, UNITED STATES

## Abstract

Diplozoidae infects the gills of cyprinid fishes in Africa, Europe, and Asia. Traditionally the hardened internal structures, crucial for identification of diplozoid species, are studied using light microscopy. Recently, the sclerotised haptoral structures of an African diplozoid, *Paradiplozoon vaalense*, were successfully isolated and visualised using scanning electron microscopy (SEM). In this paper, the haptoral sclerites of three diplozoid species are compared using SEM for the first time. *Paradiplozoon ichthyoxanthon* and *Paradiplozoon vaalense* occur on *Labeobarbus* and *Labeo* species, respectively, in the Vaal River system, South Africa, while *Diplozoon paradoxum* is widely-distributed in Europe and Asia, infecting several host species. *Diplozoon paradoxum* is a well-studied species, as well as being the type species of the family and ideal for inclusion in an exploratory study for comparative purposes. SEM study of *D*. *paradoxum* and *P*. *ichthyoxanthon* provided valuable information regarding surface morphology of the attachment structures hitherto not observed. Elaborate morphometric study of the haptoral hooks were incorporated, adding 12 point-to-point measurements to current morphometric characteristics. The results were analysed statistically, and significant differences support absolute separation (100.00%) of the three species following discriminant analysis. These point-to-point measurements could also be used for light microscopical study in the future and aid species delimitation within the Diplozoidae.

## Introduction

The Monogenea (Platyhelminthes), a widely distributed and highly diverse group of aquatic parasites, affects a substantial range of hosts. Their taxonomy is predominantly based on morphology of hardened structures in their haptoral attachment organs and reproductive systems. These structures, or sclerites, have traditionally been studied using light microscopy techniques of specimens mounted on glass slides. However, accurately describing sclerite from a 2-dimensional perspective, such as that obtained from conventional light microscopy, is limiting, particularly without sectioning and reconstruction. Some of the problems include sclerites not lying perfectly flat or overlapping. Obscured observation of structures situated deep in the parasite tissue, and sub-optimal fixing, mounting, and staining protocols provide further challenges. Maillard *et al*. [1] successfully released the male copulatory organ (MCO or cirrus) and some of the haptoral sclerites of *Diplectanum aequans* (Wagener, 1857) from their surrounding tissue using sodium carbonate, allowing these structures to be studied using scanning electron microscopy (SEM). Similar techniques were used to study the sclerites of gyrodactylid monogeneans. Mo and Appleby [2] used artificial gastric juices (HCl and pepsin) to digest the tissue of *Gyrodactylus salaris* Malmberg, 1957. Shinn *et al*. [3] combined digestive solutions (HCl–pepsin, 10% KOH, 40% Na_2_CO_3_) to release the sclerites of *Gyrodactylus* species. An inherent flaw of these techniques was the loss of some sclerites due to the differential composition of specific sclerites that resulted in inconsistent resistance to digestion. Disulphide bonds, resistant to pepsin [4] occur within some structures (cirri, hamuli, transverse bars, marginal hooks) and resist digestion to a greater extent than other, such as dorsal and ventral connecting bars. For example, Mo and Appleby [2] also studied sclerites of a larger, clamp-bearing, monogenean, *Discocotyle sagittata* (Leuckart, 1842), and obtained similar results, with only the marginal hooks being observed after digestion and the clamps being “*seen to disappear almost as fast as the soft surrounding tissue*” (*sic*.).

As an alternative to chemical or enzymatic digestion, sonication has been investigated [3] to release sclerites. But, even though all sclerites were retained, only fresh or frozen specimens could be used. Harris *et al*. [5] used proteinase K to replace the pepsin-based digestion, resulting in retention of sclerites without noticeable modification of gyrodactylid sclerites. The nature of their digestion solution also allowed for the extraction of viable genomic material from the digested tissue. More recently, authors have made use of the digestion solutions from DNA extraction kits, most of which are proteinase K based, to release the sclerites (and subsequent molecular characterisation) of several species of smaller Monogenea [6–13]. Dos Santos and Avenant-Oldewage [14] revisited the findings of Mo and Appleby [2] regarding the loss of clamp sclerites during the application of digestion techniques, and successfully observed most of the clamp sclerites of *Paradiplozoon vaalense* Dos Santos *et al*., 2015 post-digestion using SEM, while also genetically characterising these parasites.

The Diplozoidae is a fascinating group of monogeneans which have a direct lifecycle in which two diporpa larvae fuse permanently into a functional reproductive unit. This family has been recorded almost exclusively from the gills of freshwater cyprinid fishes, though several exceptions have been recorded. Diplozoids occur naturally in Africa, Asia, and Europe, although anthropogenic distribution of *Eudiplozoon nipponicum* (Goto, 1891) has resulted due to the translocation of its host *Cyprinus carpio* L. from Asia. Like most other Monogenea, the taxonomy of Diplozoidae rely heavily on the study of sclerotised structures observed using light microscopy and only recently incorporated molecular approaches. These parasites lack sclerotised genitalia and possess sclerites only in their haptor. But, due to the intricate nature of their clamp sclerites and relatively small central hooks often situated deep within the haptoral tissue, the accurate study of sclerites for taxonomic purposes remains challenging.

The success of studying sclerites post-digestion in other monogenean parasites for taxonomic purposes, as well as the findings of Dos Santos and Avenant-Oldewage [14], prompted further study of sclerites for taxonomy of diplozoids using SEM. This was done by incorporating the procedure of Dos Santos and Avenant-Oldewage [14] to study three distinct diplozoid taxa. *Paradiplozoon vaalense*, the study species of Dos Santos and Avenant-Oldewage [14], was included to confirm repeatability of results. Another distinct South African diplozoid, *Paradiplozoon ichthyoxanthon* Avenant-Oldewage, 2014, in Avenant-Oldewage *et al*. [15] from the same river system was included, as well as *Diplozoon paradoxum* von Nordmann, 1832, the genus type and most attentively studied diplozoid. *Diplozoon paradoxum* were collected from the type host *Abramis brama* (Linnaeus, 1758) to ensure similarity to type specimens.

The morphology and morphometry of the haptoral sclerites of these species were studied. The sclerite morphology of *P*. *vaalense* and *D*. *paradoxum* is well documented, but the clamp sclerites of *P*. *ichthyoxanthon* are incompletely described. Morphometric study of diplozoid sclerites are currently basic, with only the length of the hooks and handles and the length and width of the whole clamps being used. Visualisation of sclerites using SEM, provided an opportunity for more intricate morphometry. Vector- and angle-based measurements are widely applied in studies of the sclerites of other monogenean families. Thus, these approaches were modified to study the hooks of the diplozoids.

## Materials and methods

### Sample collection

*Diplozoon paradoxum* specimens were collected from *A*. *brama* in Wulpińskie Lake, Warmia and Mazury Province, Poland. Both *P*. *vaalense* and *P*. *ichthyoxanthon* were collected from their respective type hosts (*L*. *umbratus* and *L*. *aeneus*) from the Vaal Dam, Vaal River System, South Africa. Fish were euthanised by severing the spinal cord, gills removed, parasites removed, and stored in high concentrations of ethanol (above 80%).

### Digestion and SEM

**Fig 1:**
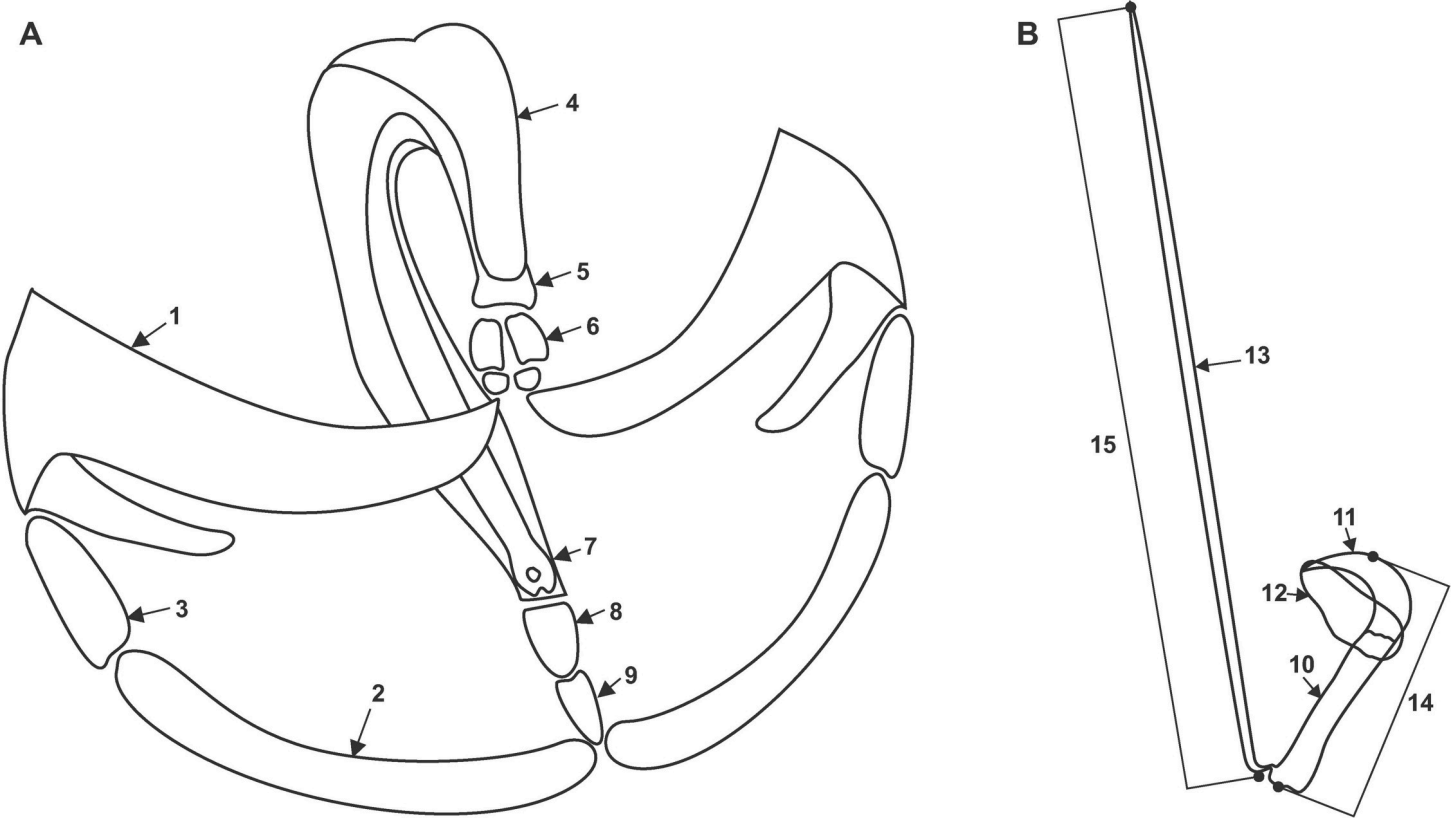
**Illustrations of the individual sclerites which make up the (A) clamps and (B) hooks of diplozoids.** (1) Anterior clamp jaw, (2) posterior clamp jaw, (3) sclerites connecting anterior end of median sclerite to clamp jaws, (4) median sclerite, (5) anterior end of median sclerite, (6) additional sclerites connecting the anterior end of the median sclerite and the anterior clamp jaws, (7) posterior end of median sclerite, (8) anterior additional sclerite, (9) posterior additional sclerite, (10) shaft, (11) blade, (12) wing, (13) handle. Measurement protocol for the (14) hook length and (15) handle length are also indicated. The length of the hook is defined as: the distance from the proximal zenith to the distal zenith of the entire hook, parallel to the inner edge of the shaft (straight part) of the hook. The length of the handle is defined as: the distance from the tip to the base of the structure.

A total of 18 diplozoid parasites, 6 of each species, were rehydrated. A haptor was removed from each adult parasite and halved. Per Dos Santos and Avenant-Oldewage [14], concavity slides (microscope slides with a depression) were used to limit the loss of sclerites. Slides were also coated with poly-L-lysine (Sigma-Aldrich, United States of America) to further immobilise freed sclerites. Each half of the haptor was placed in a concavity containing 10 μl of distilled water and 0.5 μl of tissue lysis buffer (DNeasy Blood and Tissue kit, QIAGEN Inc., Manchester, UK) was added (1 part proteinase K, 9 parts ALT tissue lysis buffer, 10 parts distilled water). Digestion times varied for individual samples, with some (usually fresher material) lysing almost instantaneously and other specimens requiring extended digestion time. On average, digestion took 5–30 minutes. Digestion endpoint was determined with a stereo microscope. Thereafter, the concavities were filled with distilled water. The lysate was removed with a micropipette and stored for molecular characterisation. Slides were dried overnight in a Sanpla dry keeper desiccator cabinet (Kita-Ku, Osaka, Japan), sputter coated with gold using an Emscope SC500 Sputter Coater (Quorum Technologies, Newhaven, U.K.) and studied using a Vega 3 LMH scanning electron microscope (Tescan, Brno, Czech Republic) at 3.4 kV. For comparative purposes, the detailed morphology of the sclerites of the three diplozoid species, as illustrated in their respective descriptions and other sources, were consulted. Standard sclerite terminology for the Diplozoidae (following Khotenovsky [16], Dos Santos and Avenant-Oldewage [14] and Owen [17]) were incorporated and adapted to discuss these structures effectively. An illustration of the components constituting the sclerites of the clamps and central hooks of diplozoids is given in Fig 1A and 1B, respectively. No protocol exists for the morphometric analyses of isolated clamp sclerites, and thus only the hook and handle were measured for comparison to existing data.

### Morphometry

**Fig 2:**
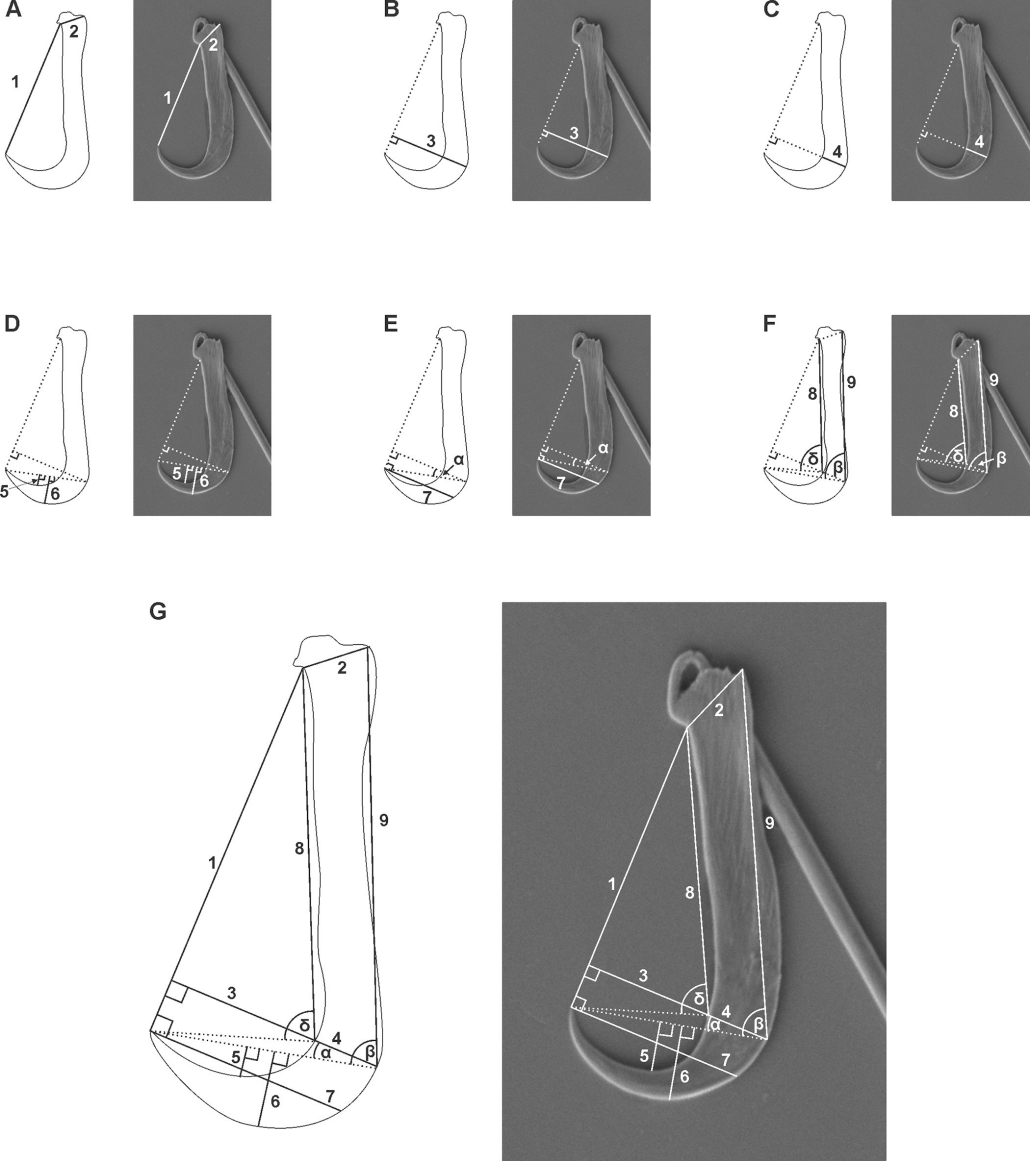
Illustrations and SEM micrographs of the hook of *Paradiplozoon vaalense* showing the morphometric parameters measured in this study. (**A**) aperture distance (AD) and proximal shaft width (PSW); (**B**) point length (PL), (**C**) distal shaft width (DSW); (**D**) inner curve length (ICL) and outer curve length (OCL); (**E**) point curve angle (PCA) and distal point length (DPL); (**F**), inner shaft length (ISL), outer shaft length (OSL), inner aperture angle (IAA) and outer aperture angle (OAA). (1) AD is defined as: the distance between the point tip and the centre of the base of the articulation of the hook and handle. (2) PSW is defined as: the distance from the base of the articulation of the hook and handle, and the zenith towards the heel of the hook. (3) PL is defined as: the perpendicular distance from the vector measuring AD to the zenith of the outer edge of the hook. (4) DSW is defined as: the measure of the width of the hook at the points where the vector describing PL pas through the structure. (5) ICL is defined as: the perpendicular distance between a vector connecting the point of the hook and the point where the vector describing PL passes through the outer edge of the hook, and the zenith of the inner edge of the blade. (6) OCL is defined as: the perpendicular distance between the vector described for ICL and the zenith of the outer edge of the blade. (7) DPL is defined as: the perpendicular distance between the end of the vector describing AD at the point of the hook and the zenith of the outer edge of the hook. (8) ISL is defined as: the distance from the point where the vector PL intercepts the inner edge of the hook, and the centre of the base of the articulation of the hook and handle. (9) OSL is defined as: the distance from the point where the vector PL intercepts the outer edge of the hook, and the point at the zenith of the heel of the hook as defined by PSW. (α) PCA is defined as: the angle between the vector describing PL and the vector connecting the point and the outer edge of the hook as defined by ICL. (β) OAA is defined as: the angle between the point at the heel of the hook as defined by PSW, the point on the outer edge of the hook as defined by PL, and the point of the hook. (δ) IAA is defined as: the angle between the centre of the base of the articulation of the hook and handle defined by AD, the point on the inner edge of the hook where PL intercepts it, and the point of the hook.

The length of the hook (HL) and handle (HNL) were measured (Fig 1B) as per convention. Following Shinn *et al*. [18] several vector and angular measurements were used to record specific morphometric characters of the hooks. Thus, 12 point-to-point measurements of the hook, similar to those used by Shinn *et al*. [18] to describe the gyrodactylid hamulus, were measured. These were: point length (PL), inner shaft length (ISL), outer shaft length (OSL), aperture distance (AD), proximal shaft width (PSW), distal shaft width (DSW), inner curve length (ICL), outer curve length (OCL), inner aperture angle (IAA), outer aperture angle (OAA), point curve angle (PCA) and distal point length (DPL), illustrated and detailed in Fig 2.

### Statistical analysis

Data were analysed by non-parametric Kruskal-Wallis *post hoc* tests to determine whether differences in single morphometric parameters could be used to separate species. Subsequently, forward stepwise linear discriminant analysis was used to analyse morphometric data. All analyses were done using SPSS v. 25 [19].

### Genetic characterisation

Extraction of DNA from the lysate was performed using a DNeasy Blood and Tissue kit following the manufacturer’s protocols. The ITS2 region was amplified using universal eukaryotic primers [20], D (5’-GGCTYRYGGNGTCGATGAAGAACGCAG-3’) and B1 (5’-GCCGGATCCGAATCCTGGTTAGTTTCTTTTCCT-3’), according to the reaction conditions of Matejusová *et al*. [21]. Successful amplification was verified in 1% GelRed (Biotuim) impregnated agarose gel and amplicons sequenced using BigDye v3.1 chemistry (Applied Biosystems) following Avenant-Oldewage *et al*. [15]. Sequencing was performed on an ABI3730 automated sequencer (Applied Biosystems). Electropherograms were inspected and edited manually (if required) using Geneious R6 [22] and run through the BLAST (NCBI) database [23] to confirm their identity. Sequence data were not published to GenBank due to the identical characteristics to available data.

### Ethical considerations

In South Africa, fish were collected after the acquisition of appropriate permits from the Gauteng Provincial Government (permit 0416; 0418; 0178; 0105; 0156). Fish were collected and killed according to the South African National Animal Ethics Guidelines and following approval by the relevant Rand Afrikaans University and University of Johannesburg Ethical committees. In Poland, fish were collected with permission from the Local University Ethics Commission of the University of Warmia and Mazury in Olsztyn (24/206-2014), under University resolution number 01/2015/D.

## Results

**Fig 3:**
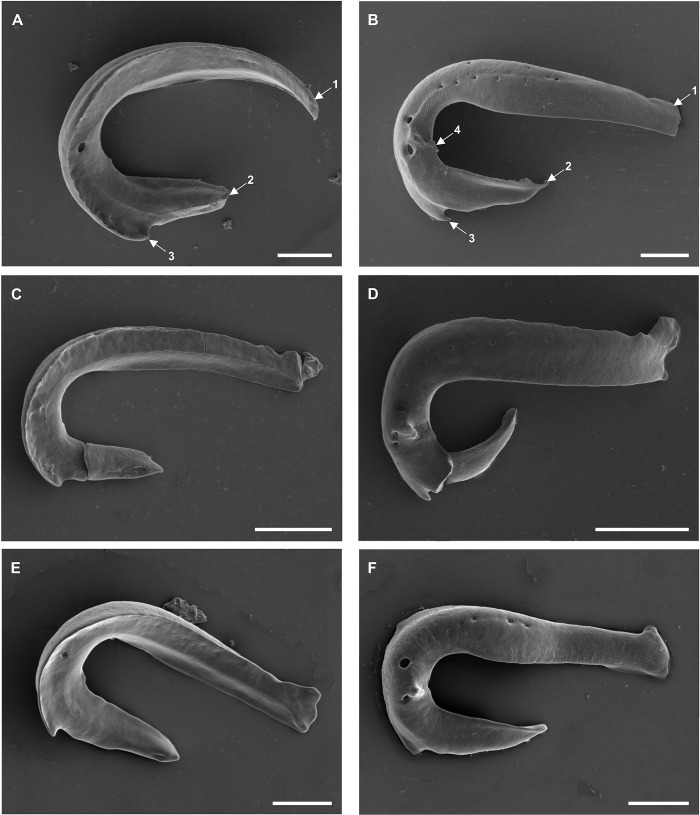
**Scanning electron micrographs of the (A, C, E) anterior and (B, D, F) posterior surface of the anterior clamp jaw of (A, B) *Diplozoon paradoxum*, (C, D) *Paradiplozoon vaalense* and (E, F) *Paradiplozoon ichthyoxanthon*.** Scale bars 20μm. (1) Proximal end, (2) distal end, (3) spur, (4) raised structure on outer surface.

**Fig 4:**
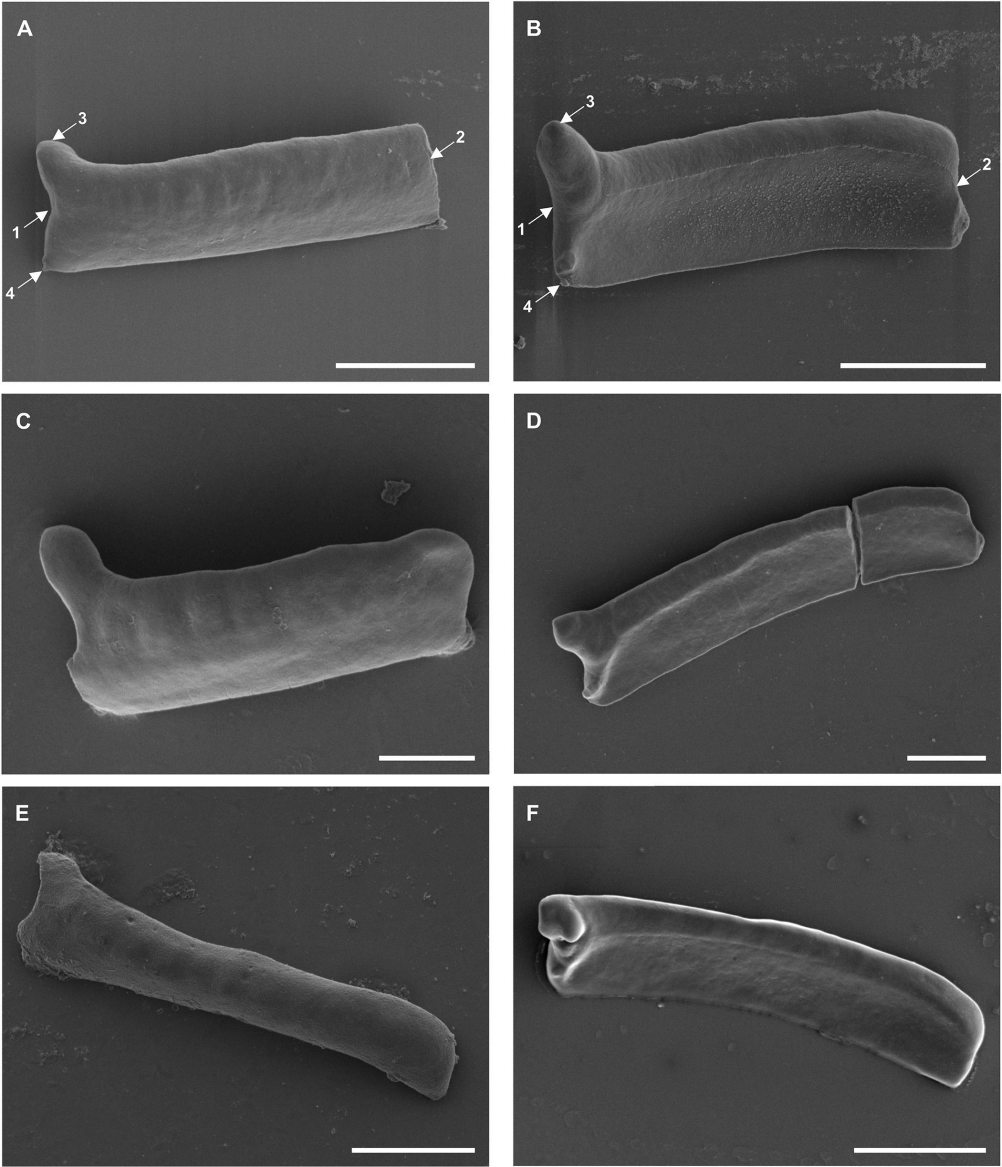
**Scanning electron micrographs of the (A, C, E) anterior and (B, D, F) posterior surface of the posterior clamp jaw of (A, B) *Diplozoon paradoxum*, (C, D) *Paradiplozoon vaalense* and (E, F) *Paradiplozoon ichthyoxanthon*.** Scale bars 20μm. (1) Proximal end, (2) distal end, (3) anterior proximal projection, (4) posterior proximal projection.

**Fig 5:**
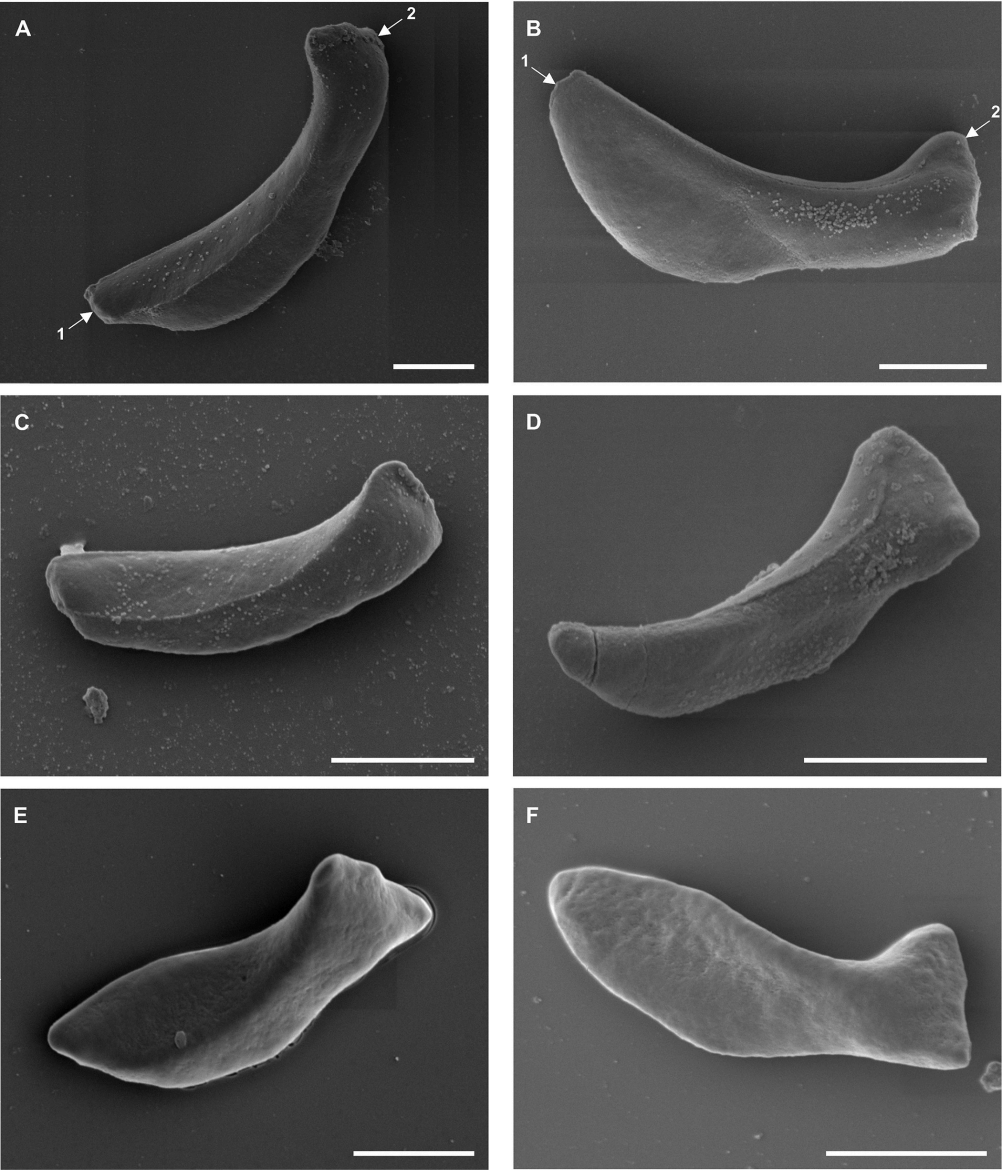
**Scanning electron micrographs of the (A, C, E) lateral and (B, D, F) central surface of the sclerite connecting anterior and posterior clamp jaws of (A, B) *Diplozoon paradoxum*, (C, D) *Paradiplozoon vaalense* and (E, F) *Paradiplozoon ichthyoxanthon*.** Scale bars 10μm. (1) Posterior, (2) anterior.

**Fig 6:**
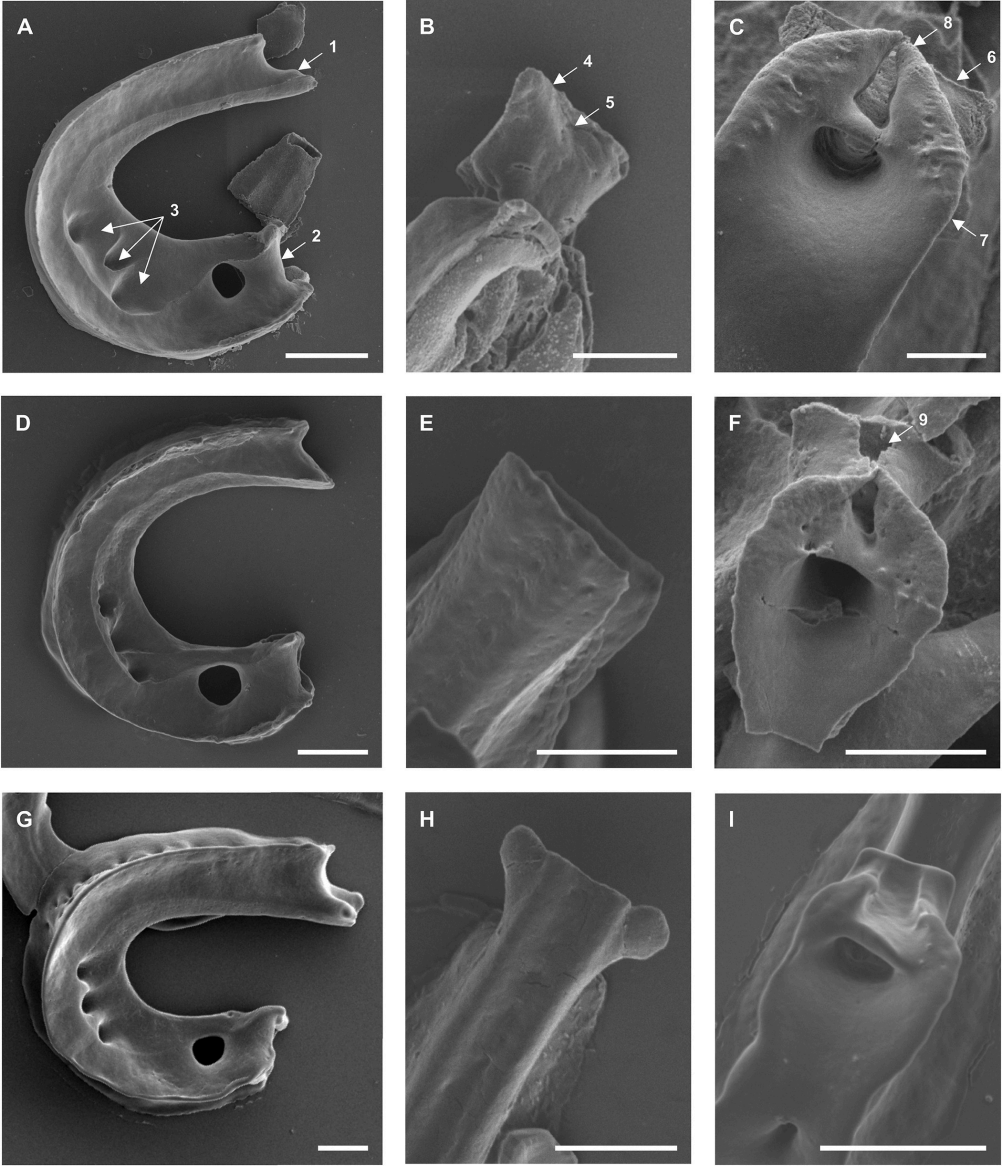
**Scanning electron micrographs of the (A, D, G) lateral view, of the (B, E, H) anterior and (C, F, I) posterior tip of the median sclerite of (A–C) *Diplozoon paradoxum*, (D–F) *Paradiplozoon vaalense* and (G–I) *Paradiplozoon ichthyoxanthon*.** Scale bars (**A**, **D**, **G**) 20μm and all other 10μm. (1) Anterior end, (2) posterior end, (3) middle fenestrations, (4) anterior projection, (5) forked anterior projection of *D*. *paradoxum*, (6) posterior projection and (7) ventral structure of median sclerite, (8) “claw”-like projection, or wings and (8) groove formed by ventral structure.

**Fig 7:**
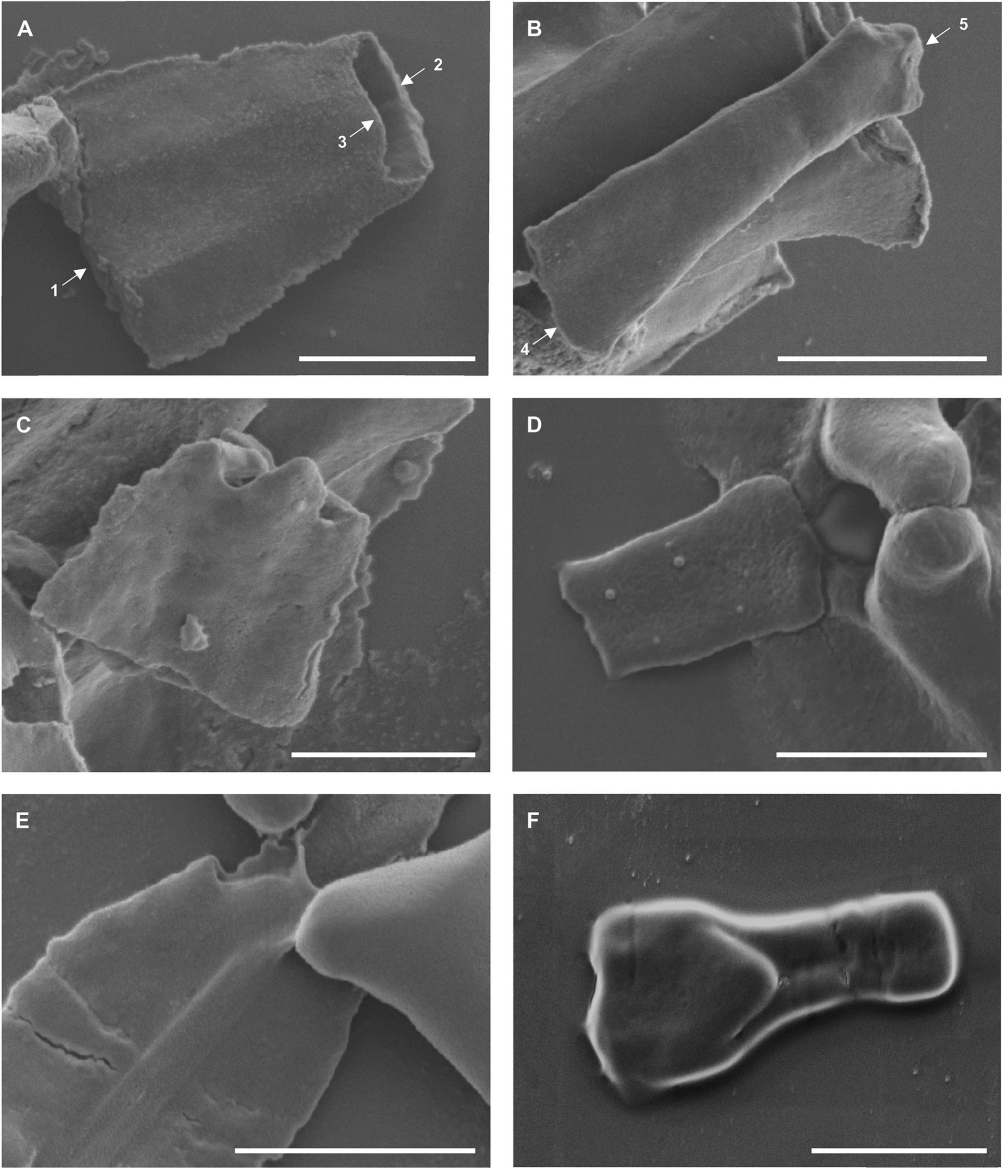
**Scanning electron micrographs of (A, C, E) anterior and (B, D, F) posterior additional sclerites of (A, B) *Diplozoon paradoxum*, (C, D) *Paradiplozoon vaalense* and (E, F) *Paradiplozoon ichthyoxanthon*.** Scale bars 10μm. (1) Anterior, (2) posterior and (3) inner posterior end of the anterior additional sclerite, (4) anterior and (5) posterior of posterior additional sclerite.

**Fig 8:**
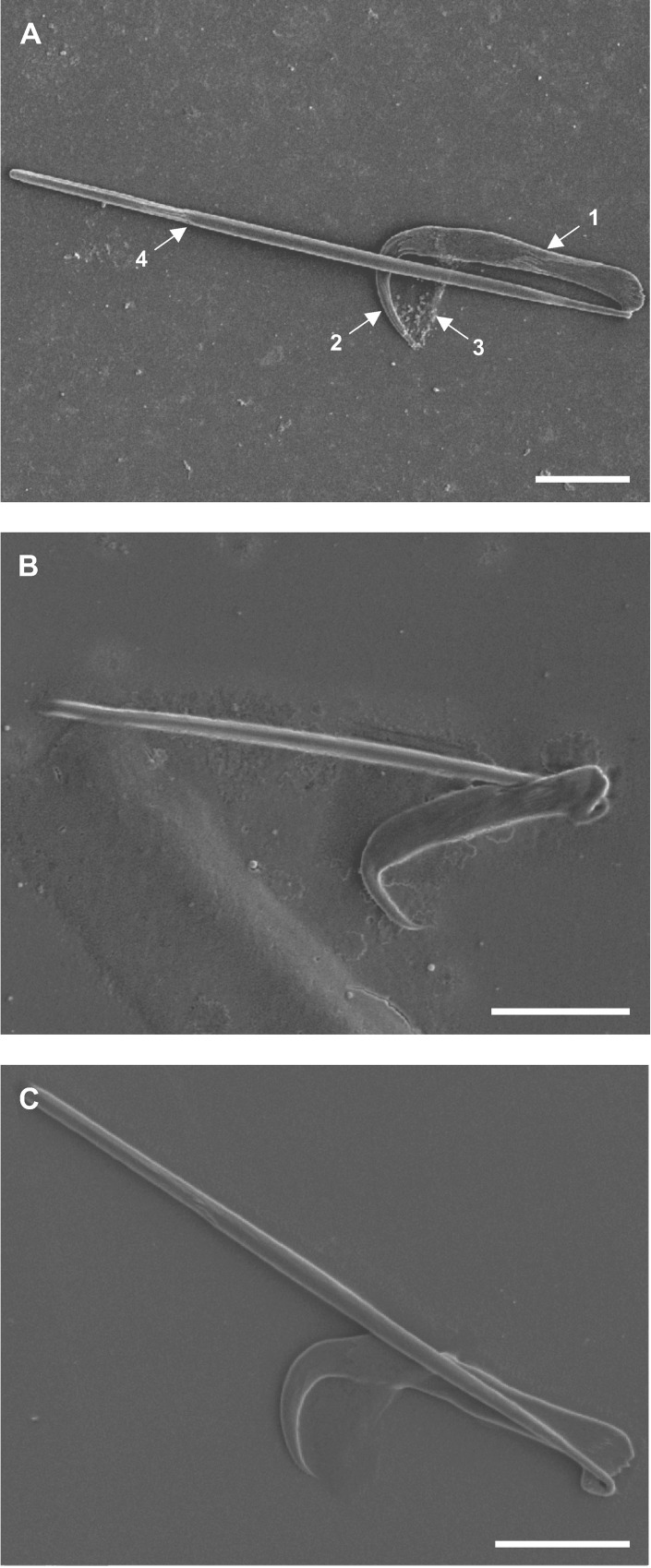
**Scanning electron micrographs of central hooks of (A) *Diplozoon paradoxum*, (B) *Paradiplozoon vaalense* and (C) *Paradiplozoon ichthyoxanthon*. Scale bars 10μm.** (1) Shaft, (2) blade, (3) wing, (4) handle.

Sclerites constituting the clamps, excluding the small sclerites connecting the anterior end of the median sclerite with the anterior clamp jaws, were successfully retained post-digestion and studied using SEM. Micrographs of the observed structures are presented in Figs 3 to 8. Consistent characteristics across the species studied, as well as recorded data, are provided below. Characters showing variation noted in Table 1.

**Table 1:** Characters of haptoral sclerites of *Paradiplozoon vaalense*, *Diplozoon paradoxum* and *Paradiplozoon ichthyoxanthon* showing marked variation when viewed post-digestion using SEM. Features of these structures noted or illustrated in previous studies are also given.

	Species					
Structure	*D*. *paradoxum*		*P*. *vaalense*		*P*. *ichthyoxanthon*	
	Previously described	Current study	Previously described	Current study	Previously described	Current study
**Anterior clamp jaw**						
Proximal end	Sharply rounded [24]*	Simple, rounded	Prominent anterior projections [25]*	More prominent Anterior projection	Rounded [15]*	Less prominent Anterior projection
Proximal region (Straight section)	Uniformly wide [24]*	Widens towards distal region (curve)	Widens towards curve [25]*	Uniformly wide	Uniformly wide [15]*	Widens towards distal region (curve)
Fenestration on inner (concave) surface at distal tip	-	Not noticeable	-	Present	-	Present
**Posterior clamp jaw**						
Distal end	Sharp [24]*	Sharp	Pointed [25]*	Rounded	Rounded [15]*	Rounded
Width	Narrow medially [24]*	Uniform	Thickened medially [25]	Stout	Uniform [15]*	Slender
Anterior surface structures	-	No structures	-	No structures	-	Several fenestrae
**Sclerites connecting the anterior and posterior clamp jaws**						
Posterior half	Long and slender [24]*	Extended distally	Uniform [25]*	Uniform	-	Widens near centre and terminates sharply
**Median sclerite**						
Central lateral fenestrae	-	Central fenestration situated notably towards the inner surface of the clamp	-	All three fenestrae equidistant from the inner surface of the clamp. Central fenestration set deeper into the sclerite and flanked by proximal and distal groves towards the inner surface of the clamp	-	All three fenestrae equidistant from the inner surface of the clamp.
Anterior end	Square [24]* Widens anteriorly [16,26] Forked [16,26]* Rounded [21,27]*	Shallowly forked	Round with trapezoid projection [25]	Truncate	Rectangular to slightly rounded [15]*	Fragile lobes at lateral the ends (Roughly diamond shaped when absent)
Ventral structure of posterior end	-	Extended wings with sharp posterior terminations	Claw-shaped extension [25]	Extended wings with sharp posterior terminations	-	Shorter wings with rounded posterior terminations
**Anterior additional sclerite**						
Inner posterior end	Rounded with possible internal structures [16,26]*	Vague projection	Rounded with possible internal structures [25]*	Prominent tri-forked appearance	-	Prominent tri-forked appearance
**Posterior additional sclerite**						
Shaft	Slender [24]*	Anterior broad, narrowing towards posterior.	Nearly rectangular [25]*	Nearly rectangular	Wide at base narrowing towards the posterior [15]*	Short and stout
Posterior end	Two individual sclerites pointed laterally [24]* Connected to posterior clamp jaws [16,26]*	Roughly diamond shaped	Rounded [25]*	Nearly rectangular	Rounded [15]*	Rounded
**Central hooks**						
Shaft	-	Long, slender	Widens towards blade [25]*	Widens towards blade	-	Straight
Point curve	-	Rounded	-	Sharp close to the shaft, almost straight towards tip, curved at the tip	Half-moon-shaped sickle with small barb [15]	Nearly rounded, slightly less so towards to tip, no barb.

* Interpreted from illustrations or images in the publication.

### Anterior clamp jaws (Fig 3)

The anterior clamp jaws of all three species have a similar shape, starting bluntly at their proximal end (where the two jaws meet), displaying a large curve, ending distally in a sharp tip, or spine. All species display a small inwardly-directed spur, although this structure is not prominent in all specimens (Fig 3A, 3C and 3F). Fenestrae are visible on the outer surface (convex) of the clamp jaw from the centre of the curved region to the midpoint of the proximal (straight) region (Fig 3B, 3D and 3F). The number and placement of the fenestrae differ between and within species, but specimens display one fenestration before and one after the raised structure centrally on the outer surface of the curve in the clamp jaws.

### Posterior clamp jaws (Fig 4)

The posterior clamp jaws of all three species are rectangular with an anterior projection towards their proximal tips (Fig 4A–4C). The outer (convex) surface of the anterior clamp jaws are smooth and frequently lack distinguishing characteristics (see Table 1). The inner (concave) surface of the clamp jaws are similar, with two proximal projections, the larger anterior and a smaller posterior, being relatively blunt and square terminally. The size of the anterior proximal projection shows a high degree of intraspecific plasticity.

### Sclerites connecting the anterior and posterior clamp jaws (Fig 5)

Sclerites connecting the anterior and posterior clamp jaws (distal-lateral sclerites) are simple structures that show little variation between the three species. This sclerite displays a sharper posterior tip and a flattened, trapezoid anterior tip.

### Median sclerite (Fig 6)

When viewed laterally, the median sclerites of all species are characteristically “U”- or “C”- shaped. This sclerite possesses a large fenestration close to the posterior tip and three smaller fenestrations in the lower part of the curved, central region of the sclerite.

The highest variation between species occur at the anterior tip of the median sclerite (Fig 6B, 6E and 6H). However, the tip of the median sclerite displays substantial variability within species, possibly due to the extent of the digestion procedure. The posterior end of the median sclerite (Fig 6C, 6F and 6I) was similar between species, with a large fenestra running through the centre, a rectangular to trapezoid posterior projection, and a rounded, “claw”-like ventral structure, or wings, on the posterior surface. The ventral structures, with their wings forming points posteriorly, appear to form a groove that follows onto the posterior projection, where it ends abruptly at the posterior tip of the projection.

### Anterior and posterior additional sclerites (Fig 7)

Additional sclerites connecting the posterior end of the median sclerite to the posterior clamp jaws were observed in all three species. The anterior additional sclerite is trapezoid, wider anteriorly (towards the medial sclerite), and narrower posteriorly. This structure appears to be hollow due to the large opening towards the posterior of the sclerites in Fig 7A and 7C. The inner posterior end displays a roundly tri-forked appearance. The posterior additional sclerite is rectangular but varies between species (see Table 1).

### Central hooks (Fig 8)

For all three species, both the hook and handle, constituting the central hook complex, were observed clearly. The shape of the hook itself varies between species, whereas the handles are not markedly different. The wing occurred intermittently, indicating its higher resistance to digestion than the parasite tissue, but not to the extent of the sclerites.

### Morphometry

In Table 2 morphometric data captured for the central hooks of *P*. *vaalense*, *D*. *paradoxum* and *P*. *ichthyoxanthon* in this study are presented. This summary includes the mean, standard deviation, and range for measurements. Raw data were used for statistical analyses.

**Table 2:** Morphometric measurements of hooks and handles of *Paradiplozoon vaalense*, *Diplozoon paradoxum* and *Paradiplozoon ichthyoxanthon* studied using SEM. Values are reported in micrometres (μm) followed by standard deviation and range (in parentheses). Number of samples = n.

	*P*. *vaalense*	*D*. *paradoxum*	*P*. *ichthyoxanthon*
	n = 10	n = 4	n = 5
HL	18.53 ± 0.39 (17.77–19.35)	30.67 ± 0.65 (30.02–31.37)	25.23 ± 0.57 (24.78–26.29)
HNL	41.45 ± 0.96 (39.23–42.07)	67.7 ± 2.34 (63.95–70.38)	55.25 ± 3.23 (51.91–59.30)
AD	12.8 ± 0.33 (12.55–13.41)	22.87 ± 0.69 (22.02–23.71)	18.08 ± 0.67 (17.65–19.40)
PSW	3.72 ± 0.26 (3.26–4.16)	3.66 ± 0.15 (3.42–3.80)	4.10 ± 0.28 (3.66–4.53)
PL	7.95 ± 0.30 (7.36–8.44)	13.08 ± 0.31 (12.62–13.49)	10.54 ± 0.11 (10.39–10.71)
DSW	2.81 ± 0.22 (2.61–3.18)	3.38 ± 0.21 (3.16–3.69)	3.85 ± 0.18 (3.59–4.12)
ICL	1.89 ± 0.18 (1.67–2.10)	3.37 ± 0.20 (3.05–3.60)	2.53 ± 0.16 (2.42–2.85)
OCL	3.06 ± 0.27 (2.80–3.52)	4.85 ± 0.28 (4.42–5.22)	4.34 ± 0.20 (4.00–4.59)
PCA	13.43 ± 2.81 (9.44–17.81)	9.49 ± 2.28 (7.50–13.15)	15.46 ± 3.95 (10.68–21.54)
ISL	12.08 ± 0.36 (11.31–12.83)	22.81 ± 0.58 (22.22–23.76)	16.59 ± 0.54 (15.63–17.22)
IAA	85.13 ± 5.45 (76.57–92.98)	77.76 ± 3.44 (74.30–83.17)	89.69 ± 4.72 (84.71–96.61)
OSL	15.94 ± 0.41 (15.05–16.75)	25.34 ± 0.51 (24.75–26.08)	21.03 ± 0.34 (20.55–21.48)
OAA	78.7 ± 3.44 (73.72–84.57)	75.35 ± 3.00 (72.55–80.39)	79.91 ± 3.29 (76.49–85.84)
DPL	7.46 ± 0.45 (6.70–8.06)	12.77 ± 0.29 (12.39–13.12)	10.03 ± 0.18 (9.86–10.37)

Significance of the non-parametric test are shown in Table 3. The majority of the 14 point-to-point measurements supported discrimination between the three species. Only three characteristics appear to be redundant: PSW, OAA, and PCA, while IAA, does not support significant discrimination between *P*. *vaalense* and *P*. *ichthyoxanthon*.

**Table 3:** Morphometric characters of the central hooks of *Paradiplozoon vaalense*, *Diplozoon paradoxum* and *Paradiplozoon ichthyoxanthon* showing significant variation between species based on the Kruskal–Wallis *post hoc* test (P<0.05). For abbreviations see *Morphometry* section of the methods section.

	*P*. *vaalense*	*P*. *ichthyoxanthon*
*D*. *paradoxum*	HL	HL
	HNL	HNL
	PL	PL
	ISL	ISL
	OSL	OSL
	AD	AD
	DPL	DPL
	DSW	DSW
	ICL	ICL
	OCL	OCL
	IAA	IAA
*P*. *vaalense*	-	HL
		HNL
		PL
		ISL
		OSL
		AD
		PSW
		DPL
		DSW
		ICL
		OCL

**Fig 9:**
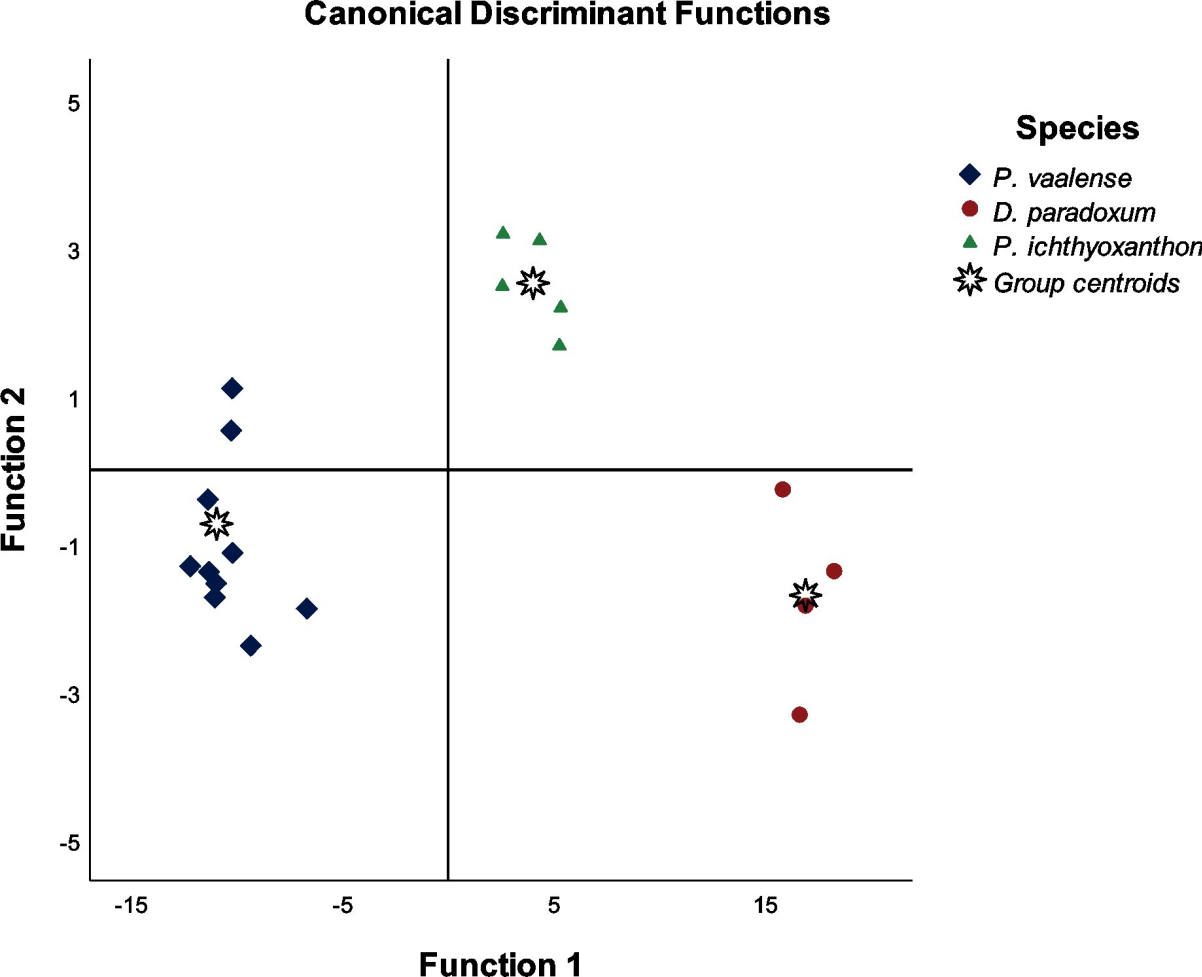
Plot of the canonical discriminant functions based on the best morphometric variables selected by a forward discriminant analysis of the measurements obtained from the hooks of *Diplozoon paradoxum*, *Paradiplozoon vaalense* and *Paradiplozoon ichthyoxanthon*.

Discriminant analysis based on 14 point-to-point measurements showed significant mean differences for twelve characteristics. These were: hook length, outer shaft length, inner shaft length, point length, aperture distance, distal point length, handle length, inner curve length, outer curve length, inner aperture angle and proximal shaft width. The remaining characteristics (point curve angle and outer aperture angle) were not significant for species discrimination. Results for the tests of equality of means is given in Table 4. However, only three variables were identified as significant predictors for the structure matrix (Table 5). The classification results of the discriminant analysis showed 100.00% of specimens were correctly identified (Table 6), as indicated in the plot (where the data group distinctly for individual species) of the canonical discriminant functions in Fig 9.

**Table 4:** Tests of equality of group means for 14 point-to-point measurements produced by discriminant analysis to distinguish between *Paradiplozoon vaalense*, *Diplozoon paradoxum* and *Paradiplozoon ichthyoxanthon*. Significance with P>0.05 in bold.

	Wilks' Lambda	F	df1	df2	Sig.
Hook length	0.011	654.033	2	14	0.000
Outer shaft length	0.013	529.564	2	14	0.000
Inner shaft length	0.013	518.585	2	14	0.000
Point length	0.017	406.653	2	14	0.000
Aperture distance	0.017	404.210	2	14	0.000
Distal point length	0.026	258.525	2	14	0.000
Handle length	0.040	167.860	2	14	0.000
Inner curve length	0.080	80.006	2	14	0.000
Outer curve length	0.086	74.587	2	14	0.000
Distal shaft width	0.155	38.031	2	14	0.000
Inner aperture angle	0.552	5.679	2	14	0.016
Proximal shaft width	0.626	4.177	2	14	0.038
Point curve angle	0.668	3.473	2	14	**0.060**
Outer aperture angle	0.779	1.989	2	14	**0.174**

**Table 5:** Structure matrix produced through discriminant analysis of 14 point-to-point measurements of the hooks of *Paradiplozoon vaalense*, *Diplozoon paradoxum* and *Paradiplozoon ichthyoxanthon*. Correlations were pooled within-groups between discriminating variables and standardized canonical discriminant functions.

	Function	
	1	2
Hook length	0.774*	0.084
Handel length	0.392*	-0.034
Distal shaft width	0.129	0.913*
Inner aperture angle^b^	0.181	0.287*
Distal point length^b^	0.082	-0.145*
Proximal shaft width^b^	0.515*	0.233
Outer aperture angle^b^	0.267*	0.094
Point curve angle^b^	0.159*	0.051
Outer curve length^b^	0.066*	0.002
Aperture distance^b^	0.459*	-0.052
Outer shaft length^b^	0.551*	-0.061
Point length^b^	0.215*	-0.142
Inner curve length^b^	0.242*	-0.168
Inner shaft length^b^	0.322*	-0.250

* Largest absolute correlation between each variable and any discriminant function

^b^ This variable not used in the analysis.

**Table 6:** Classification results based on stepwise discriminant analysis of the morphometric characters of the central hooks of *Paradiplozoon vaalense*, *Diplozoon paradoxum* and *Paradiplozoon ichthyoxanthon*. Original and cross-validated group cases were 100.00% correctly classified.

			Predicted Group Membership			
		Species	*P*. *vaalense*	*D*. *paradoxum*	*P*. *ichthyoxanthon*	Total
Original	Count	*P*. *vaalense*	8	0	0	8
		*D*. *paradoxum*	0	4	0	4
		*P*. *ichthyoxanthon*	0	0	5	5
	%	*P*. *vaalense*	100.0	.0	.0	100.0
		*D*. *paradoxum*	.0	100.0	.0	100.0
		*P*. *ichthyoxanthon*	.0	.0	100.0	100.0
Cross-validated	Count	*P*. *vaalense*	8	0	0	8
		*D*. *paradoxum*	0	4	0	4
		*P*. *ichthyoxanthon*	0	0	5	5
	%	*P*. *vaalense*	100.0	.0	.0	100.0
		*D*. *paradoxum*	.0	100.0	.0	100.0
		*P*. *ichthyoxanthon*	.0	.0	100.0	100.0

### Molecular findings

Sufficient genomic DNA was obtained from the digestion of half a haptor, allowing for successful amplification and sequencing of the ITS2 rDNA fragment. Sequences obtained from the same haptor (from each half) and those from the same species were identical, and thus produced three haplotypes. These haplotypes were identical to published sequence data for *D*. *paradoxum* (accession numbers AF369759; AJ563372; KP326299), *P*. *ichthyoxanthon* (accession number HF566124), and *P*. *vaalense* (accession number HG423142), respectively. Thus, the identity of the parasites was confirmed.

### Remarks

Because Dos Santos and Avenant-Oldewage [14] were involved in both studies, comparisons can be made between the techniques used. As per Dos Santos and Avenant-Oldewage [14], the use of concavity slides greatly assisted in retaining sclerites and in the efficiency of the technique. The use of poly-L-lysine-coated slides, as compared to non-coated concavity slides, did not make a substantial difference to the results obtained. The diluted digestion buffer (1:1 buffer to water in initial solution) proved as successful as the concentrated buffer. Due to the relatively large size of diplozoid haptors, they could be split and processed separately, providing both SEM and molecular material. As in Dos Santos and Avenant-Oldewage [14], some of the smaller sclerites were not observed, and the identification of poorly-understood sclerites post-digestion was challenging, due to the clamp not retaining its composite state.

## Discussion

### Comparison of results to previous work

The exposed sclerites of *P*. *vaalense* observed using SEM, here and by Dos Santos and Avenant-Oldewage [14], complement the diagram of the clamps in the species description, regarding not only the general shapes of the sclerites, but also the intricate anterior and posterior ends of the median sclerite. The similarity in the morphology of the clamp and central hook sclerites, produced in both SEM studies of *P*. *vaalense*, reinforce the reliable, repeatable nature of this method. However, unlike in Dos Santos and Avenant-Oldewage [14], the posterior additional sclerites were also observed in this study. The shape of these sclerites differ from the bullet-shaped illustration in Dos Santos *et al*. [25], suggesting that the application of this technique improves sclerite observation (see Table 1).

The sclerites of *P*. *ichthyoxanthon* have hitherto been studied almost exclusively using conventional light microscopy. The illustration of clamp sclerites in the description of *P*. *ichthyoxanthon* lack detailed taxonomic information required for proper species differentiation. The results presented here greatly improve the resolution of the clamp sclerite detail. In particular, the anterior end of the median sclerite. The anterior and the posterior additional sclerites were observed clearly, differing greatly to the single, elongated structure illustrated in Avenant-Oldewage *et al*. [15].

Although the sclerites of *D*. *paradoxum* have been studied predominately using conventional light microscopy, sectioning and reconstruction have been used as well [17,28]. While the description and illustration of the clamp sclerites of *D*. *paradoxum* are detailed, they do not display or describe all morphological characteristics of importance for taxonomy. Considering recent illustrations of clamp detail of *D*. *paradoxum*, the results obtained here support the taxonomic information available for this species.

Measurements of the hooks and handles of all three species did not overlap and could easily be distinguished. The standard deviation for the hooks did not exceed 0.70 μm, while the deviation of the handles did not exceed 3.30 μm (see Table 2). The lengths observed in this study are within the ranges of previously recorded data (see Table 7).

**Table 7:** Ranges of measurements (in μm) obtained in the current study alongside those reported in previous publications for the central hooks of *Paradiplozoon vaalense*, *Diplozoon paradoxum* and *Paradiplozoon ichthyoxanthon*.

	Hook length	Handle length
*D*. *paradoxum*		
Current study	30.02–31.37	63.95–70.38
Khotenovsky [16]	28.00–33.00	58.00–71.00
*P*. *vaalense*		
Current study	17.77–19.35	39.23–42.07
Dos Santos *et al*. [25]	18.00–20.00	39.00–49.00
*P*. *ichthyoxanthon*		
Current study	24.78–26.29	51.91–59.3
Avenant-Oldewage *et al*. [15]	25.00*	53.00*

* Only mean values given.

### General morphology of sclerites

Owen [17] (later Bovet [28] and Khotenovsky [16]) produced highly detailed, although schematic, diagrams of the clamp sclerites of *D*. *paradoxum* to elucidate details of their structure and mechanisms. In doing so, Owen [17] represented the basic characteristics of diplozoid clamps thoroughly, while comparing the clamp to that of *Discocotyle sagittata* (the species studied by Mo and Appleby [2]). Owen [17] noted the hollow nature and the small, inwardly-directed spur in the distal region of the anterior clamp jaws. Septa-like partitions in the hollow spaces of these clamp jaws were recorded by Goto [29], and the multitude of fenestrae on the outer and inner surfaces of the clamp jaws support this. From a functional point of view, the results presented here form an image coherent with the findings of Owen [17]. The author noted that, unlike that of the corresponding sclerite in *Discocotyle*, the sclerite connecting the anterior and posterior clamp jaws (distal-lateral sclerite) moves in a definite socket on the curved region of the anterior jaws. The raised structure on the outer surfaces of the anterior jaws in Fig 3A, 3C and 3E may be the location of this socket. Owen [17] also indicated the presence of an intrinsic adductor muscle attaching these two sclerites close to the socket, possibly explaining the concentration of fenestrae at this point. However, as can be seen in Fig 5, no clear attachment site for a muscle can be seen on the connecting sclerites.

Owen [17] paid specific attention to the posterior tip of the median sclerite and the additional sclerites connecting it to the posterior clamp jaws. The author described the transition of extrinsic longitudinal muscles, arising anteriorly to the clamps, which join to form a slender tendon passing through the aperture piercing the posterior end of the median sclerite. This aperture, or fenestra (seen in Fig 6) and the deep groove along the outer surface of the median sclerite, essentially leading to this opening, supports the attachment of this tendon. Owen [17] further concluded that this tendon passes over the inner surface of the anterior additional sclerite, passing through a perforation, then along the outer edge of the posterior additional sclerite, and finally bifurcates and enters the posterior edge of the tip of the proximal ends of the posterior clamp jaws. From the posterior clamp jaws shown in Fig 4, no opening for tendons to enter is visible, but the small posterior projection observed may be the attachment site of these units. The inner opening of the aperture through the median sclerite forms a channel-like structure posteriorly, supporting the observations of Owen [17]. The anterior additional sclerites in Fig 7A and 7C show a shallow groove on their inner surface, possibly housing the tendon. In Fig 7E, a delicate structure appears to be lying on top of the inner surface of this sclerite, which may be the remnants of the tendon in question. When considering the outer surface of the anterior additional sclerite of *P*. *vaalense* depicted in Dos Santos and Avenant-Oldewage [14], in conjunction with posterior tips of the wings of the ventral structure on the posterior tip of the median sclerite forming a channel-like structure continuing towards the posterior projection of this sclerite, it appears that some functional unit runs along this groove, then along the outer surface of the anterior additional sclerite, and through the aperture formed at its posterior end.

### Diplozoid sclerite study

Since the description of *D*. *paradoxum*, the morphology, interpretation, and detail of the haptoral sclerites given in descriptions has fluctuated. The clamp description produced by von Nordmann [24] displayed beautifully-illustrated structures consisting of several separate sclerites in a functional unit. Goto [29] stated that the clamps of *E*. *nipponicum* were not as intrinsically complex as noted by von Nordmann [24]. Goto [29] only notes five sclerites, in comparison to the 18 noted by von Nordmann [24]. Goto [29] thoroughly described the structure of the clamps of *E*. *nipponicum* through sectioning, and noted the hollow nature of the clamp jaws and the deep “cut” in the posterior wall of the median sclerite. However, the illustration of the clamps by Goto [29] was minimalistic compared to that of von Nordmann [24]. As the study of the Diplozoidae progressed, a generalised clamp morphology was revealed consisting of a pair of anterior and a pair of posterior clamps jaws, sclerites allowing for the articulation of these two units, a curved median sclerite and additional sclerites between the anterior and posterior end of this median sclerite connecting them to their respective clamp jaws (Fig 1A). However, the descriptions of most diplozoid taxa display varying complexities of clamp sclerite detail, with some presenting simpler structures, as per Goto [29] (e.g. [30,31]), while others produced more detailed illustrations (e.g. Gläser [32]).

The most prominent hurdle in diplozoid taxonomy is the lack of robust morphological criteria for discriminating between taxa at species level. Gläser and Gläser [33] noted that the size of the central hook and the shape of the clamps were the most effective and reliable characters for the identification of diplozoid taxa. Both criteria suffer from intrinsic flaws. As the central hooks are simple sclerites, their measurement is easy, but only if they are viewed in a perfectly horizontal orientation. If not, the measurements are skewed and may lead to incorrect descriptions or identifications. Similarly, the shape of the clamps themselves suffer from the plasticity of these attachment organs. Depending on the orientation of observation, the state in which the specimen was fixed, the amount of pressure applied to the specimen during preparation, and the effect of various mounting mediums, the shape and size of the clamps can be substantially distorted. Thus, the possibility of studying the haptoral sclerites of diplozoids, as confirmed by Dos Santos and Avenant-Oldewage [14], may offer solutions to these problems.

As most of the components of the general diplozoid clamp (as per Khotenovsky [16]) were observed post-digestion, it may suggest that these are the only sclerites found in the clamps. This would suggest that the observations of both von Nordmann [24] and Goto [29], were flawed. However, the additional sclerites connecting the anterior end of the median sclerites and the anterior clamp jaws were not observed. This shows that there is still room for improvement of digestion techniques, as these additional sclerites were either lost, overlooked, or succumbed to digestion (possibly having a similar composition to the wing of the central hook). The central hooks were distinct and reinforce the convention of studying both the hook and the handle as a unit [29], although these two structures are easily separated due to the delicacy of their articulation [24].

Currently, only the lengths and widths of the clamps are measured. This is not possible after digestion, as the clamps lose their composite structure and only the individual sclerites are observed. The central hooks on the other hand are much simpler to study after digestion as they only consist of two simple structures, the hook and handle. Even when these two structures are severed from one another, they can still be measured. However, only the total length of these structures has been used in diplozoid morphometry. Within other groups of monogeneans, like the smaller gyrodactylid worms, the measurement and quantitative analyses of sclerotised structures is well-established. The sclerites, especially the haptoral sclerites, of these worms are simpler structures, similar to the hooks of the Diplozoidae, and thus easier to flatten and study accurately using light microscopy. This allows for both mounted and digested material to be assessed similarly and directly compared, making several multivariate analyses (such as principal component analyses) possible.

As such, the addition of another 12 point-to-point measurements were defined for diplozoid hooks (based on the variables described by Shinn *et al*. [18]), bringing the total characters used to 14. Analyses of these 14 variables through non-parametric tests and tests of equality of group means derived from discriminant analysis indicated at least 10 of the 14 variables could be used to accurately distinguish *D*. *paradoxum*, *P*. *vaalense* and *P*. *ichthyoxanthon*. This means that the additional measurements investigated here could be used to discriminate between other diplozoid species, possibly allowing for the clarification of taxonomic uncertainty between morphologically similar species. This is further supported by the 100% accuracy of correct species identification by discriminant analysis and the clear, distinct grouping of data according to different species when a plot of canonical discriminant functions (Fig 9) is constructed. These point-to-point measurements could also be used to study the hooks of diplozoids using conventional light microscopy.

Other aspects that need to be addressed before the SEM study of diplozoid sclerites can be optimised, include the determination of the true intraspecies variability of certain features, such as the fenestrae. There appears to be a clear species-specific differentiation in the appearance of the three fenestrae on the central lateral surfaces of the median sclerite, while other fenestrae are either similar in all three species or vary greatly within species. Khotenovsky [16] suggested that the characteristics of the fenestrae in diplozoids may be a taxonomically important feature, but this has not been practically addressed until now. However, to support this hypothesis, a much larger sample size, with more species and a further refined technique, is needed to promote accurate analyses of sclerites post-digestion. This may even allow the sclerites connecting the median sclerites and the anterior clamps jaws to be retained. This technique should also be compared to other proposed methods, such as laser scanning confocal fluorescence microscopy, which have shown promising results in the study of other monogenean families, although not readily accessible to some researchers.
